# Development and Validation of the Brief Nursing Stress Scale (BNSS) in a Sample of End-of-Life Care Nurses

**DOI:** 10.3390/nursrep11020030

**Published:** 2021-04-30

**Authors:** Noemí Sansó, Gabriel Vidal-Blanco, Laura Galiana

**Affiliations:** 1 Department of Nursing and Physiotherapy, University of the Balearic Islands, 07122 Palma, Spain; Noemi.Sanso@uib.es; 2 Department of Nursing, University of Valencia, 46010 Valencia, Spain; Gabriel.Vidal@uv.es; 3 Department of Methodology for the Behavioral Sciences, University of Valencia, 46010 Valencia, Spain

**Keywords:** stress, burnout, job satisfaction, nurses, end-of-life care

## Abstract

Nursing has been identified as a very stressful profession. Specifically in end-of-life care, nurses frequently experience stressful situations related to death and dying. This study aims to develop and validate a short scale of stress in nurses, the Brief Nursing Stress Scale. A cross-sectional survey of Spanish end-of-life care professionals was conducted; 129 nurses participated. Analyses included a confirmatory factor analysis of the Brief Nursing Stress Scale, estimation of reliability, relation with sex, age and working place, and the estimation of a structural equation model in which BNSS predicted burnout and work satisfaction The confirmatory factor analysis showed an adequate fit: *χ*^2^(9) = 20.241 (*p* = 0.017); CFI = 0.924; SRMR = 0.062; RMSEA = 0.098 [0.040,0.156]. Reliability was 0.712. Women and men showed no differences in stress. Younger nurses and those working in hospital compared to homecare showed higher levels of stress. A structural equation model showed nursing stress positively predicted burnout, which in turn negatively predicted work satisfaction. Nursing stress also had an indirect, negative effect on work satisfaction. The Brief Nursing Stress Scale showed adequate estimates of validity, reliability, and predictive power in a sample of end-of-life care nurses. This is a short, easy-to-use measure that could be employed in major batteries assessing quality of healthcare institutions.

## 1. Introduction

Nursing has been generally identified as a very stressful profession, ‘by its very nature’ [1], and prevalence of occupational stress and burnout has been repeatedly stated [2,3]. In Spain, studies have pointed high levels of emotional exhaustion in nurses, and moderate levels of depersonalization [4,5].

Stress is an important cause of job dissatisfaction in nursing [6] and can cause job leaves [7]. It is worth highlighting that occupational stress among nurses usually decreases their efficiency of job performance, with a consequently negative effect on the quality of patient care and patient satisfaction [8,9]. Occupational stress has been related to staff conflicts, absenteeism, decreased productivity, lowered morale, and burnout, among others [10]. The immediate responses of the human body to occupational stress and burnout can be physiological, psychological, and behavioral. Stressful situations also have devastating consequences at the psychosomatic level, such as headaches, fatigue, nausea, skin rash, and weight fluctuations [11]; and several psychological symptoms have also been related to stress situations, including anxiety, nervousness, tension, depression, and irritation [12].

Research on nursing stress has pointed out several risk factors defining such stress, including working conditions like low job control and high job demands, being moved among different patient care units within the organization, being short of essential resources, and having low supportive work relationships with co-workers, supervisors, and/or physicians [13,14,15,16,17,18], together with personal variables, such as neuroticism and emotional coping [5]. These causes of stress are common to the nursing work environment and have been found in all specialties [15].

Specifically, in the palliative care context, nurses frequently experience stressful situations related to death and dying. Nursing providing end-of-life care face additional stressful and demanding situations, such as caring with scientific technical knowledge, decision-making related to ethical issues, and constant contact with suffering, end of life, and death of people for whom they care [19,20]. These challenges usually cause physical, psychological, and emotional distress as well as work-related stress, which can lead to the development of burnout [21,22].

Thus, some sources of stress identified as key in the nursing literature, both at a general level and in end-of-life care, include [23]: (1) stressful situations derived from the process of dying or death; (2) stressful situations derived from conflicts with physicians; (3) stressful situations derived from lack of support; (4) stressful situations derived from conflict between nurses; (5) stressful situations derived from workload; and (6) stressful situations derived from uncertainty of the treatment.

Due to its importance for the development of burnout and the well-being of nurses, but also due to the consequences on patients’ health and the quality of care, the measurement of stress in nursing is key for health institutions. As pointed, stress can cause job dissatisfaction [6], job leaves [7], and even the consumption of addictive substances [24]. However, the stressful conditions to be measured in the healthcare context are enormous, and having to answer infinite questionnaires makes the workload of the nurses even greater, limiting their valuable time. For these reasons, it is very important to have brief measures in place to screen and detect potential sources of stress. And then, only then, apply longer batteries, which allow us to deepen our understanding of these conditions and try to solve them.

To respond to these circumstances, the present study aims to develop and validate a short scale of stress in nurses, the Brief Nursing Stress Scale (BNSS). For this purpose, we will present the scale, study its internal structure, gather reliability evidence, and quantify its predictive power over burnout and work satisfaction.

## 2. Materials and Methods

### 2.1. Development of the Brief Nursing Stress Scale

The Brief Nursing Stress Scale (BNSS) is based the dimensions of stress pointed in the Nursing Stress Scale (NSS) [25], which included: (1) stressful situations derived from the process of dying or death; (2) stressful situations derived from conflicts with doctors; (3) stressful situations derived from lack of support; (4) stressful situations derived from conflict between nurses; (5) stressful situations derived from workload; and (6) stressful situations derived from uncertainty of the treatment. This scale is one of the most popular and widely used instrument to assess stressors in nursing [23] and was originally developed based on the psychological model of stress described by Lazarus [26] and Appley and Trumbull [27].

Taking into account the six stressors already pointed, two experts in nursing and methodology turned the dimensions into the six final items that composed the instrument. For example, if the dimension was “stressful situations derived from the process of dying or death”, the item corresponding to the dimension specifically asked for “how frequently you suffer stressful situations derived from the process of dying or death”. This procedure was used for the six dimensions. The sentences were rated according to agreement, using a Likert-type, 4-point scale, from 1 (never) to 4 (almost always); therefore, using the original scale of the Spanish version of the Nursing Stress Scale [28]. Total score was calculated with the mean of the scores in the six items, and ranged from 1 to 4. Item content can be consulted in Table 1.

### 2.2. Design, Setting, and Participants

A cross-sectional survey of Spanish end-of-life care professionals was conducted to assess variables influencing professionals’ compassionate care. This cross-sectional study has been reported using the Strengthening the Reporting of Observational Studies in Epidemiology (STROBE) Statement [29].

The survey was conducted during January–February 2020. Professionals were encouraged to participate through the Spanish Society for Palliative Care (SECPAL). Participants were sampled from their lists of members, who were asked to complete an online survey using SurveyMonkey, a secure and anonymous online platform that also restricted multiple survey responses. Participation was voluntary and required respondents’ informed consent.

For inclusion, the participants had to be a healthcare professional (physician, nurse, psychologist, nursing assistant, social worker, or others), who currently cared for patients at the end of their lives, but not necessarily in palliative care settings.

The sample consisted of 296 end-of-life care professionals who answered the survey, including nurses, physicians, psychologists, social workers, etc. Specifically for this study, the subsample of 129 nurses was selected. Following Wolf, Harrington, Clark and Miller’s [30] work, one-factor, six-indicator model with loadings of 0.50, 0.65, and 0.80 required sample sizes of 90, 60, and 40, respectively [30]. According to this work, sample size was sufficient to detect medium factor loadings.

### 2.3. Variables

Sociodemographic variables included age, sex, and place of work. Other variables measured were:

- Workload, measured with the item “I have too much workload”, ranging from 0 (never) to 3 (almost always).
- Work control, measured with the item “I have control over my workload”, ranging from 0 (never) to 3 (almost always).
- Burnout, measured with the Maslach Burnout Inventory—Human Services Survey (MBI) [31]. This is a 22-item questionnaire that relates to three constructs of burnout: emotional exhaustion (9 items), depersonalization (5 items), and personal accomplishment (8 items). Each item rated on a seven-point Likert-type scale for how frequently they experience the feeling, from 0 (never) to 6 (every day).
- Work satisfaction, measured with the General Work Satisfaction Scale from the Michigan Organizational Assessment Scale [32]. The scale is composed by three items. Each item rated on a five-point Likert-type scale, from 1 (completely disagree) to 5 (completely agree).

### 2.4. Analyses

First of all, descriptive statistics were calculated for the items of the scale, including means and standard deviations. Additionally, means and standard deviations of the total score in the Brief Nursing Stress Scale, burnout dimensions, and work satisfaction were also calculated.

Second, and in order to study the factorial structure of the BNSS, a one-factor confirmatory factor analysis (CFA) model was hypothesized, estimated, and tested. Model fit was assessed using the following statistic and fit indexes: the chi-square, the Comparative Fit Index (CFI), the Root Mean Squared Error of Approximation (RMSEA) and the Standardized Root Mean Squared Residuals (SRMR) index. Adequate fit is generally assumed with CFI > 0.90 together with a RMSEA/SRMR < 0.08, while values of CFI/TLI > 0.95 and RMSEA/SRMR < 0.05 indicate excellent fit [33]. The method of estimation for the CFA was Weighted Least Squares Mean and Variance corrected (WLSMV), given the ordinal nature of the data [34].

Reliability was estimated using the Composite Reliability Index.

*t* tests for independent samples were used to study differences between women and men, and between home and hospital workers. Nursing stress relation with age, workload, and work control was studied using Pearson correlations.

Finally, the predictive power of nursing stress, as measured with the BNSS, over burnout and work satisfaction was assessed using a full structural equation model. Specifically, nursing stress was hypothesized to predict burnout, which in turn predicted work satisfaction. The three constructs, nursing stress, burnout, and work satisfaction, were modeled as latent factors, and consequently, free of error of measurement. In order to assess model fit, the fit criteria mentioned above were used.

SPSS version 24 [35] was used to estimate descriptive statistics, *t* tests, and Pearson correlations. MPLUS version 8.4 [36] was used to estimate the CFA and the full structural equation model.

### 2.5. Ethical Considerations

The study was approved by the Ethics Research Committee at the University of the Balearic Islands (82CER18). Given the characteristics of the study, the people who decided to participate voluntarily were told the reason and purpose for carrying out the work. This entire study complied with the ethical principles for research in health sciences established at the national and international levels in the Declaration of Helsinki [37]. Special attention was paid to confidentiality and protection of privacy, guaranteeing the anonymity of the information provided, which was used exclusively for this work and was held in the custody of the research team. In addition, our research team is committed to strictly complying with the Organic Spanish Law on Personal Data Protection, which guarantees that the participants in this study can exercise their rights of access, rectification, cancellation, and opposition to the collected data.

## 3. Results

### 3.1. Participants Description

Mean age was 43.5 years old (SD = 10.63); 84.5% (*n* = 109) were women. 44.2% (*n* = 57) were hospital workers, 39.5% were home care workers, and 16.3% (*n* = 21) worked in other facilities, such as elderly institutions or hospices.

### 3.2. Descriptive Statistics

BNSS items showed medium levels in the six domains of nursing stress, with means ranging from 1.96 (item 3, stressful situations derived from lack of support) to 2.74 (item 5, stressful situations derived from workload) (see Table 2). The total score of the scale was 2.36.

Regarding the rest of the variables, levels of workload were high, work control was medium, emotional exhaustion was moderate, depersonalization was low, and personal acceptance and work satisfaction were high.

### 3.3. Internal Structre and Reliability

The CFA showed an adequate fit, except for the RMSEA: *χ*^2^(9) = 20.241 (*p* = 0.017); CFI = 0.924; SRMR = 0.062; RMSEA = 0.098 [0.040,0.156]. Based on Kenny et al.’s results [38], the overall fit was considered good. Factor loadings were adequate, ranging from 0.338 (item 2) to 0.710 (item 4). Details can be consulted in Table 2.

Reliability of the BNSS was adequate, with CRI = 0.712.

### 3.4. Relations between Nursing Stress, Sex, Age, Working Place, Workload, and Work Control

The *t* test for independence samples showed no statistically significant differences in nursing stress between women and men: *t*(127) = 1.027; *p* = 0.307. The correlation between nursing stress and age showed a negative, statistically significant relation (*r* = −0.181; *p* = 0.044), although of small value. Regarding the study of the relationship between nursing stress and working place, the *t* test was statistically significant: *t*(106) = −2.683; *p* = 0.008. Hospital nurses showed higher levels of stress (M = 2.47; SD = 0.44) compared to homecare nurses (M = 2.26; SD = 0.35).

### 3.5. Prediction of Burnout and Work Satisfaction

Nursing stress was related to burnout and work satisfaction. Specifically, we hypothesized, estimated, and tested a structural equation model, in which nursing stress directly predicted burnout and indirectly predicted work satisfaction, an effect mediated by burnout.

**Figure 1:**
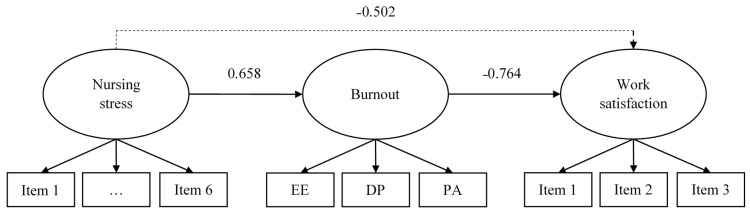
Structural equation modeling predicting burnout and work satisfaction. Notes: EE = Emotional exhaustion; DP = Depersonalization; PA = Personal acceptance. All the factor loadings, direct effects and the indirect effect were statistically significant (*p* < 0.001). Script line represents the indirect effect.

The model showed an adequate fit: *χ*^2^(52) = 93.854 (*p* < 0.001); CFI = 0.925; SRMR = 0.084; RMSEA = 0.079 [0.053,0.104]. As regards the analytical fit, and as can be seen in Figure 1, nursing stress positively predicted burnout, which in turn negatively predicted work satisfaction. Nursing stress also had an indirect, negative effect on work satisfaction. Overall, more than 40% of burnout variance was explained (*R*^2^ = 0.432; *p* < 0.001), and almost 60% of work satisfaction (*R*^2^ = 0.584; *p* < 0.001).

## 4. Discussion

The aim of the study was to develop and test the psychometric properties of the Brief Nursing Stress Scale, a short measure of nursing stress, in a sample of end-of-life care nurses. The scale, composed by six items that represented the six original dimensions of the Nursing Stress Scale [25], presented adequate evidence of reliability and validity.

The results of this study are similar with a previous study that showed a high level of occupational stress among cancer care nurses [39]. Although Kim & Kim [39] used a different scale to measure stress, they found that the dimension related to excessive workload was the most affected, as was in the present study. To be aware of such stress levels is of great importance due to the well-known association of stress with reduced work performance, higher job turnover, decreased job satisfaction, loss of productivity, high rates of absenteeism, and reduced quality of nursing care for patients [40,41,42,43].

In addition, our results pointed estimates of adequate internal validity, supporting the appropriateness of the one-factor structure of the BNSS scale. Its six items were loaded into a single dimension of nursing stress. Reliability was also adequate.

Regarding age, the results of this study sample show a negative statistically significant relation between nursing stress and age, confirming previous studies [44]. Nevertheless, the results obtained in the current study do not fully coincide with previous research, where the greatest stress was found among those aged 40 or over, followed by those in the age group of 25 to 39 years and under 25 years [39]. Additionally, no gender differences were found. In the literature, results on the relation between age and gender are not clear. Whereas there are some studies which found a positive association between female gender and stress [45,46,47,48], some others found that male nurses are at greater risk [49,50]. Talking about the association of stress with the working place, the great stress found among hospital nurses over homecare nurses is not coincident with previous research, such as Martens, where no differences were found among different work places [51].

Finally, we tested a structural equation model in which nursing stress explained both burnout syndrome and job satisfaction. Nursing stress, as measured with the BNSS, showed evidence of test-criterion validity, being a strong, direct predictor of burnout syndrome, and indirectly related to job satisfaction. This is in line with previous research, which had already pointed how nursing stress can produce high levels of burnout [18,52,53,54], and low levels of job satisfaction [54,55].

Limitations of the study are mainly referred to the sample size. Furthermore, some shortcomings include the lack of test-retest reliability, due to the study cross-sectional nature. Other limitation is the absence of information regarding the public or private ownership of the centers, or the cities in which professionals worked. These limitations could be addressed in future works in which the BNSS could be used in bigger samples, longitudinal studies, or other cultural contexts. Among its strengths is its briefness. This scale could be used as a quick, screening tool to detect stressful situations in the working environment.

## 5. Conclusions

All in all, evidence gathered in this study has shown adequate estimates of validity, reliability, and predictive power of the Brief Nursing Stress Scale in a sample of end-of-life care nurses. Based on the well-known and widely recognized model of Nursing Stress Scale, the BNSS is a short, easy-to-use measure that could be employed in major batteries assessing quality of healthcare institutions, with adequate prediction capacity of problems of burnout and job satisfaction.

## Figures and Tables

**Table 1:** The Brief Nursing Stress Scale (BNSS).

Item Number	Item Content Please Indicate How Frequently You Suffer…
1	stressful situations derived from the process of dying or death
2	stressful situations derived from conflicts with doctors
3	stressful situations derived from lack of support
4	stressful situations derived from conflict between nurses
5	stressful situations derived from workload
6	stressful situations derived from the uncertainty of the treatment

**Table 2:** Descriptive statistics for the Brief Nursing Stress Scale (BNSS) items, total score, indicators of workload and work control, dimensions of burnout, and work satisfaction.

Variable	Mean	SD ^1^	Minimum	Maximum	λ
Item 1	2.60	0.65	1.00	4.00	0.347
Item 2	2.35	0.67	1.00	4.00	0.338
Item 3	1.96	0.61	1.00	4.00	0.664
Item 4	2.35	0.67	1.00	4.00	0.710
Item 5	2.74	0.75	1.00	4.00	0.478
Item 6	2.16	0.67	1.00	4.00	0.666
Nursing stress	2.36	0.40	1.33	3.67	---
Workload	2.91	0.78	1.00	4.00	---
Work control	2.44	1.03	1.00	4.00	---
Emotional exhaustion	17.53	8.68	2.00	45.00	---
Depersonalization	4.55	3.67	0.00	19.00	---
Personal acceptance	33.30	6.22	14.00	48.00	---
Work satisfaction	4.36	0.75	1.67	5.00	---

^1^ Standard deviation.

## Data Availability

The data that support the findings of this study are available from the corresponding author upon reasonable request.
